# GAUSS - genesis of asteroids and evolution of the solar system: A sample return mission to Ceres

**DOI:** 10.1007/s10686-021-09800-1

**Published:** 2021-10-15

**Authors:** Xian Shi, Julie Castillo-Rogez, Henry Hsieh, Hejiu Hui, Wing-Huen Ip, Hanlun Lei, Jian-Yang Li, Federico Tosi, Liyong Zhou, Jessica Agarwal, Antonella Barucci, Pierre Beck, Adriano Campo Bagatin, Fabrizio Capaccioni, Andrew J. Coates, Gabriele Cremonese, Rene Duffard, Manuel Grande, Ralf Jaumann, Geraint H. Jones, Esa Kallio, Yangting Lin, Olivier Mousis, Andreas Nathues, Jürgen Oberst, Holger Sierks, Stephan Ulamec, Mingyuan Wang, The GAUSS Team

**Affiliations:** 1 Max Planck Institute for Solar System Research, Göttingen, Germany; 2 Present Address: Shanghai Astronomical Observatory, Shanghai, China; 3 NASA Jet Propulsion Laboratory, La Cañada Flintridge, CA USA; 4 Planetary Science Institute, Tucson, AZ USA; 5 School of Earth Sciences and Engineering, Nanjing University, Nanjing, China; 6 Institute of Astronomy and Space Science, National Central University, Chung Li, Taiwan; 7 School of Astronomy and Space Science, Nanjing University, Nanjing, China; 8 Istituto Nazionale di AstroFisica – Istituto di Astrofisica e Planetologia Spaziali (INAF-IAPS), Rome, Italy; 9 Institute for Geophysics and Extraterrestrial Physics, Technical University Braunschweig, Braunschweig, Germany; 10 LESIA-Observatoire de Paris, Université PSL, CNRS, Université de Paris, Sorbonne Université, F-92195 Meudon, Principal Cedex, France; 11 CNRS Institut de Planétologie et d’Astrophysique, Univ. Grenoble Alpes, Grenoble, France; 12 Universidad de Alicante, Departamento de Física, Ingeniería de Sistemas y Teoría de la Señal, Alicante, Spain; 13 Mullard Space Science Laboratory, University College London, Surrey, UK; 14 INAF - Osservatorio Astronomico di Padova, Padova, Italy; 15 Instituto de Astrofísica de Andalucía (CSIC), Granada, Spain; 16 University of Aberystwyth, Aberystwyth, UK; 17 Institute of Geological Sciences, Free University of Berlin, Berlin, Germany; 18 School of Electrical Engineering, Aalto University, Aalto, Finland; 19 Institute of Geology and Geophysics, Chinese Academy of Sciences, Beijing, China; 20 Aix Marseille Univ, CNRS, CNES, LAM, Marseille, France; 21 DLR Institute of Planetary Research, Berlin, Germany; 22 DLR Space Operations and Astronaut Training, Cologne, Germany; 23 National Astronomical Observatory, Chinese Academy of Science, Beijing, China

**Keywords:** Ceres, Dwarf planet, Sample return, Ocean world, Habitability, Voyage 2050

## Abstract

The goal of Project GAUSS (Genesis of Asteroids and evolUtion of the Solar System) is to return samples from the dwarf planet Ceres. Ceres is the most accessible candidate of ocean worlds and the largest reservoir of water in the inner Solar System. It shows active volcanism and hydrothermal activities in recent history. Recent evidence for the existence of a subsurface ocean on Ceres and the complex geochemistry suggest past habitability and even the potential for ongoing habitability. GAUSS will return samples from Ceres with the aim of answering the following top-level scientific questions:

- What is the origin of Ceres and what does this imply for the origin of water and other volatiles in the inner Solar System?
- What are the physical properties and internal structure of Ceres? What do they tell us about the evolutionary and aqueous alteration history of dwarf planets?
- What are the astrobiological implications of Ceres? Is it still habitable today?
- What are the mineralogical connections between Ceres and our current collections of carbonaceous meteorites?

## Background

Though the Rosetta mission to comet 67P/Churyumov-Gerasimenko came to an end only recently, in 2016, it is important to recall that the planning activity eventually leading to its approval by ESA was initiated more than three decades earlier in 1983. An equally, if not more, ambitious project in the framework of “Voyage 2050” is proposed here. The target is the innermost dwarf planet, Ceres, which was discovered on New Year’s Day of 1801, by the Italian astronomer Giuseppe Piazzi at Palermo Observatory. At the time of its discovery, Ceres was considered to be the missing planet between the orbits of Mars and Jupiter as predicted by the Titius-Bode law [38]. Ceres’ location was confirmed in December the same year using the orbital elements calculated by then 24-year-old Carl Friedrich Gauss [111]. The name of the project with the acronym of GAUSS for “Genesis of Asteroids and evolUtion of the Solar System” is partly a tribute to this scientific episode of great importance in astronomy and planetary science.

## Scientific rationale

### Ceres in the history of the solar system

The formation of the oldest solids found in the Solar System – calcium-aluminium inclusions (CAIs) – dates back 4567.5 Myr [22]. After that, the formation of gas giants must finish before the dispersal of the protoplanetary gas disc, which lasts 2-10 Myr [4, 46]. Then, about 30-100 Myr later, the terrestrial planets formed in the planetesimal disc [50].

The planetesimal disc not only provided all the materials that constitute the terrestrial planets and cores of gas giants, but also exerted perturbing forces on the grown planets [40, 55]. This perturbation might have continuously modified the planetary orbits in a relatively gentle way [32]. Before the dispersal of the gas disc, planets could have already experienced migration due to the interaction with the massive gas disc [118]. During the planetary migration afterwards, mechanisms such as low-order mean motion resonance between planets brought up abrupt variations in the orbital configuration of planets [115]. The interaction between the planetesimal disc and migrating planets/protoplanets reconfigured the structure of the Solar System [36, 70, 118]. However, the growth and evolution of terrestrial planets and asteroids (in a wide radial range from Mars’ orbit to the Kuiper belt) in the early stages of the Solar System are not fully understood.

Given that their properties depend on their formation circumstances and evolutionary processes experienced since their formation, small bodies (e.g., asteroids, comets, and trans-Neptunian objects) in the Solar System are of interest to researchers because they provide a way to probe the protoplanetary disc from which our Solar System formed [6, 52, 103]. By determining the chemical and physical properties of various small bodies in our Solar System, we can gain insight into chemical and thermal conditions in different areas of the protoplanetary disc and also investigate the chemical, thermal, collisional, and dynamical processes that have shaped those populations since their formation. A successful scenario of planet migration should provide the mechanisms that were able to efficiently deliver planetesimals from different zones to the main belt. In addition, the structure of the main belt, the mass depletion, the proper excitation of orbital eccentricities and inclinations, should all be reproduced.

**Fig. 1:**
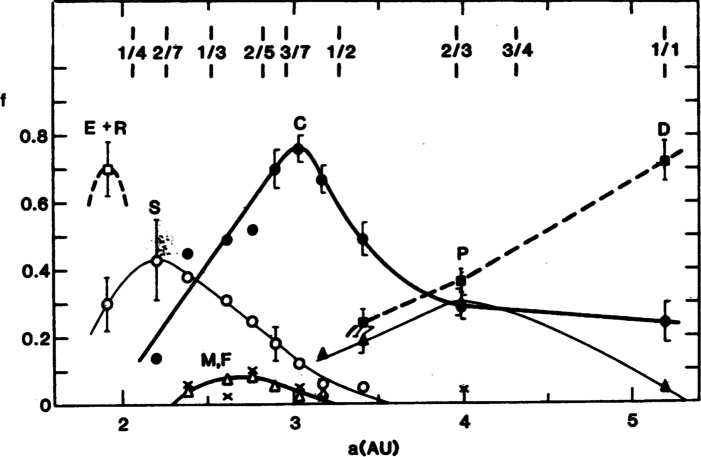
Relative distribution of taxonomic types for a bias-corrected sample of 656 main-belt asteroids. Letters indicate different compositional types. Refer to Table 1 in [37] for the definition of each type. Locations of major resonances with Jupiter are indicated at the top. (Figure reprinted from [37])

One of the earliest attempts to map the compositional structure of our asteroid belt and use it to infer information about the origin and evolution of the Solar System was performed by [37]. They found a systematic distribution of compositional types of asteroids in the main asteroid belt that they suggested was consistent with chemical condensation models of the Solar System (Fig. 1). The authors concluded that it was unlikely that this distribution could be explained by the chaotic transport of objects from different regions of the Solar System into the asteroid belt, and proposed instead that it indicated that the asteroids formed at or near their present locations.

The work of [37] has since undergone a major update in the form of a study by [27], who used the Sloan Digital Sky Survey (SDSS) Moving Object Catalog data to derive taxonomic classifications for about 35000 objects, including objects as small as 5 km in diameter. The inclusion of much smaller objects in this sample than were previously available, as well as the computation of compositional distributions by mass (instead of by number, see Fig. 2 in [28]) suggest that the asteroid belt preserves a history of Solar System evolution that is far more complex than previously thought [28, 36, 63, 118], with asteroids of various taxonomic types scattered throughout the asteroid belt, including in regions where they are not expected based on dynamically static Solar System formation models. Instead, the history of the Solar System as recorded by main-belt asteroids likely includes relatively brief periods of dramatic mixing caused by giant planetary migration, e.g., those proposed as parts of the well-known Nice and Grand Tack models [63, 64, 70, 115, 115, 118] as well as less dramatic but ongoing processes such as collisions and small body migration due to mean-motion resonances with the giant planets and the Yarkovsky effect (e.g., [7, 8, 31, 35, 88]).

**Fig. 2:**
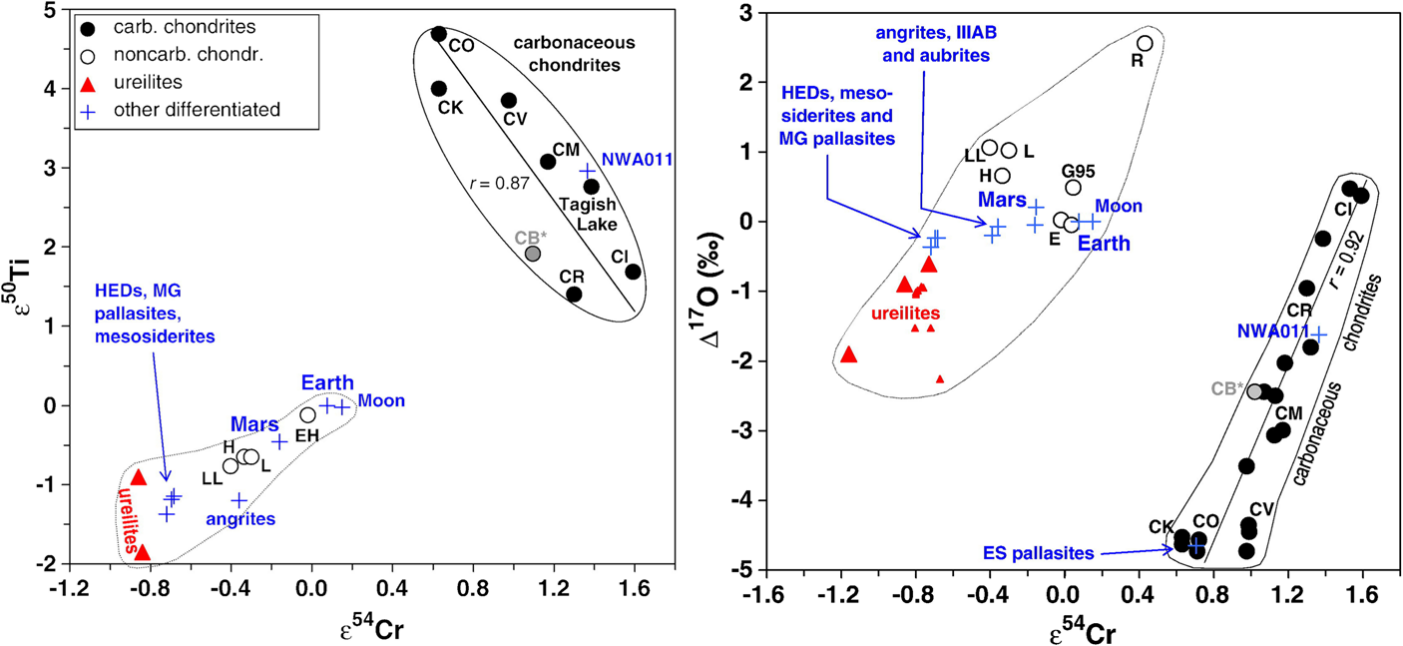
Dichotomy of stable isotopes among planetary materials. (Figure reprinted from [119])

The fraction and composition of ice in a body is of particular interest due to the strong temperature constraints they provide. The so-called “snow line” refers to the distance from the Sun at which the temperature dips below the condensation temperature of water, causing it to freeze into solid ice and then become incorporated into accreting planetesimals. The exact location of the snow line in our protoplanetary disc was dependent on a variety of poorly constrained environmental conditions including opacity, mass density, and accretion rate in the disc, and is also thought to have shifted with time as planetesimal accretion progressed and the aforementioned properties of the disc changed [71].

Bodies formed in the outer Solar System such as between Jupiter and Neptune (the original accretion zone of current Oort Cloud objects; [40, 120]) and beyond the orbit of Neptune (the Kuiper Belt) are well beyond the snow line. Closer to the Sun, the situation is less certain. Observations of asteroids suggest that the snow line probably existed as close as 2.5 au from the Sun [37, 52], but theoretical studies [19, 62, 97] have placed it as close in as the orbit of Mars, or even closer. If this is true, it means that objects throughout the main asteroid belt could have incorporated some water ice at the time of their formation.

Evidence of past and present-day ice has in fact been found in main-belt asteroids. Studies of meteorites linked to the asteroid belt as well as remote spectroscopic observations of asteroids have revealed the presence of aqueously altered minerals, indications that liquid water was once present [47, 53, 90]. Meanwhile, spectroscopic evidence of water ice frost has been reported for various main-belt asteroids including (24) Themis [41, 89, 110], while some main-belt objects have even been observed to exhibit comet-like activity that has been attributed to the sublimation of present-day volatile ices, i.e., the so-called main-belt comets [48]. The location, abundance, distribution, and inferred water content of currently and formerly icy main-belt objects provide valuable clues for discerning the primordial location and evolution of the snow line, although this of course is also complicated by the aforementioned likely transport of at least some small bodies from their original formation locations due to giant planet migration and other dynamical processes.

The bulk-rock isotope anomalies of meteorites reveal an isotopic dichotomy: two distinct trends defined by the non-carbonaceous (NC) bodies and the carbonaceous (CC) bodies respectively (Fig. 2). The CC bodies include carbonaceous chondrites, a few ungrouped achondrites, and IIC, IID, IIF, IIIF, IVB iron meteorites. The NC bodies include Earth, the Moon, Mars, ordinary chondrites, enstatite chondrites, and most of achondrites. This dichotomy has been further observed in Mo, W, Ru, and Ni isotopic systematics [11, 58, 72, 80]. However, the origins of the isotopic anomalies and their correlations are unknown, although ^54^Cr-enrichment can be attributed to nano-sized Cr-oxide probably ejected from supernovas [83]. Based on the isotopic dichotomy, [119] proposed that the CC bodies formed in the outer Solar System and the NC bodies formed in the inner Solar System. The formation of Jupiter blocked the material exchange between the two groups. As a consequence of radial migration and mass growth of the giant planets in the Solar System, some CC planetesimals and embryos moved inwards from the outer Solar System to the main belt [58, 88, 118, 119].

Ceres, the largest object in the main belt, preserves many clues to the formation and evolution of the main belt, as well as of the Solar System. The surface material of Ceres is similar to carbonaceous chondrite, though no meteorite has so far been linked with Ceres [91]. Interpretation of the data from the Dawn mission with geochemical simulations show that the surface mineralogy of Ceres is consistent with the aqueous alteration of CI and CM chondrite [15].

The ammonia detected on Ceres [23] suggests that Ceres could have originated in the outer Solar System, and migrated to its current neighbourhood. However, this scenario still misses definitive evidence. Ceres could have formed in the outer Solar System or formed in situ and accreted materials radially transported from the outer Solar System [68]. The stable isotope anomalies of samples from Ceres could provide ground truth on its formation location (Fig. 2). Furthermore, geochemical information in returned samples could be used to understand the evolution path of Ceres.

### Results from the dawn mission

The Dawn mission performed a 3.5 years rendezvous with Ceres starting from early 2015, mapping its geomorphology at resolutions down to ∼35 m globally [92], derived surface mineralogy at resolutions down to 140 m [33], the top layer (∼0.5-1 m) elemental abundance at effective resolutions of tens of km, as well as the gravity field to 18 degrees of spherical harmonics[56]. During the last mission phase, Dawn entered a long elliptical orbit with an altitude of 35 km at periapsis for a detailed study of the narrow longitudinal strip at about 240^∘^ E that stretches from north of Occator crater and goes up to Azacca crater on the other hemisphere. During this phase, images were acquired with resolutions as high as 3.5 m/pixel.

The Dawn results confirmed that Ceres is volatile-rich, has a partially differentiated interior, and has experienced global aqueous alteration. Dawn revealed Ceres as a geologically active dwarf planet with brine-driven volcanism as recently as of a few Myr and probably even at present. Ammoniated phyllosilicates are distributed all over the surface of Ceres [3, 23]. The incorporation of ammonia in Ceres’ surface mineralogy is an indication that Ceres may have accreted materials from the giant planet formation region.

The surface layer of Ceres is rich in water of hydration and water ice. The abundance of water ice is relatively low towards the equator and high towards the polar regions [81]. The latitudinal variation is consistent with the evolution of subsurface water ice as controlled by the thermal condition on the surface and shallow subsurface of Ceres [99, 100] and the low obliquity of Ceres [96]. Water ice has been directly identified in about ten specific places on the surface, near rim shadows in fresh craters at latitudes poleward of 30^∘^ [20, 21], and inside the permanently shadowed craters in the polar regions [79, 101]. The existence of water ice in the first few km of the crust is also inferred by analyzing lobate morphologies [98].

**Fig. 3:**
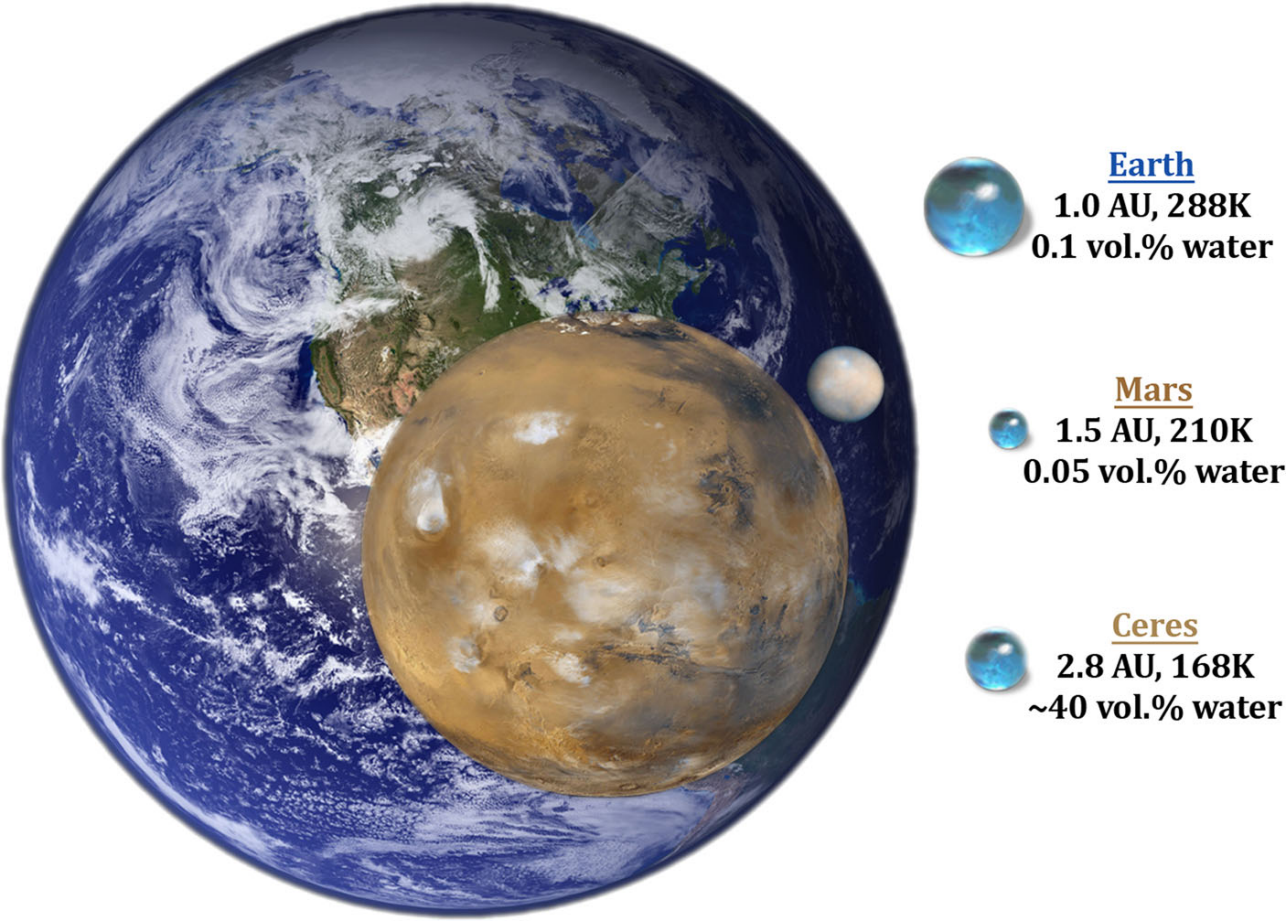
Comparison view of water abundances on Earth, Mars, and Ceres

The abundant water ice on Ceres (Fig. 3) led to differentiation and aqueous alteration that shaped the mineralogical composition of Ceres in its crust and mantle [15]. Near-infrared data returned by the Visible and InfraRed mapping spectrometer (VIR) on board the Dawn spacecraft detected carbonates and ammonium salts that are previously found only on Earth and Enceladus [24]. The existence of brines at depth played a key role in driving the geological process on Ceres, including volcanism and activities in its geologically recent history [94, 95, 105].

**Fig. 4:**
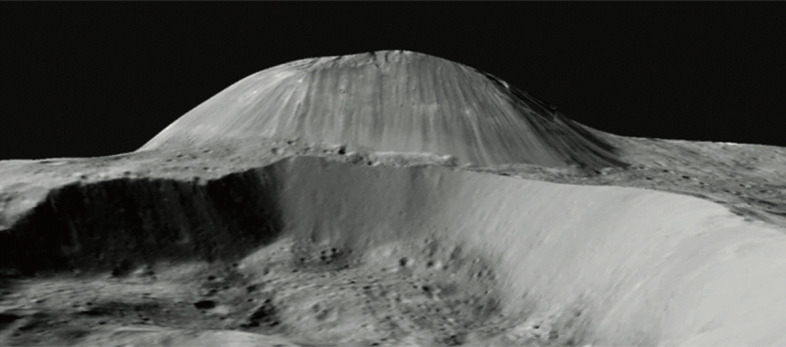
Perspective view of Ahuna Mons on Ceres from Dawn Framing Camera data (no vertical exaggeration). The mountain is 4 km high and 17 km wide in this south-looking view (image credit: NASA/JPL/Caltech/IAPS/MPS/DLR/INAF/ASI)

The most prominent geomorphological feature that is considered of volcanic origin is Ahuna Mons (Fig. 4) [94, 95]. Its distinct size, shape, and morphology are consistent with being a volcanic dome formed by extrusions of highly viscous melt-bearing material. At the summit is the buildup of a brittle carapace, which partially fractured and disintegrated to generate the slope debris. The bright streaks in the slope debris are rich in Na-carbonates [12, 123, 124]. The gravitational relaxation of the enclosed ductile core shapes the overall topographic profile of the summit, requiring an extruded material of high viscosity. The age of the most recent activity on Ahuna Mons is about 210 ± 30 Myr [94]. Other possible features involving the presence of melt include smaller domes [108, 109], fractures in the crater floor [9], post-impact modification by the deposition of extended plains material with pits and widely dispersed deposits that form a diffuse veneer on the preexisting surface [57].

Shallow subsurface volatiles are also evident from the many other geomorphological features, such as the pitted terrain [107]. On the other hand, the crater morphology and the simple-to-complex crater transition indicate that Ceres’ outer shell is likely neither pure ice nor pure rock, but a mixture of ice, rock, salts and/or clathrates that allows for limited and spatially variable viscous relaxation [5, 45].

**Fig. 5:**
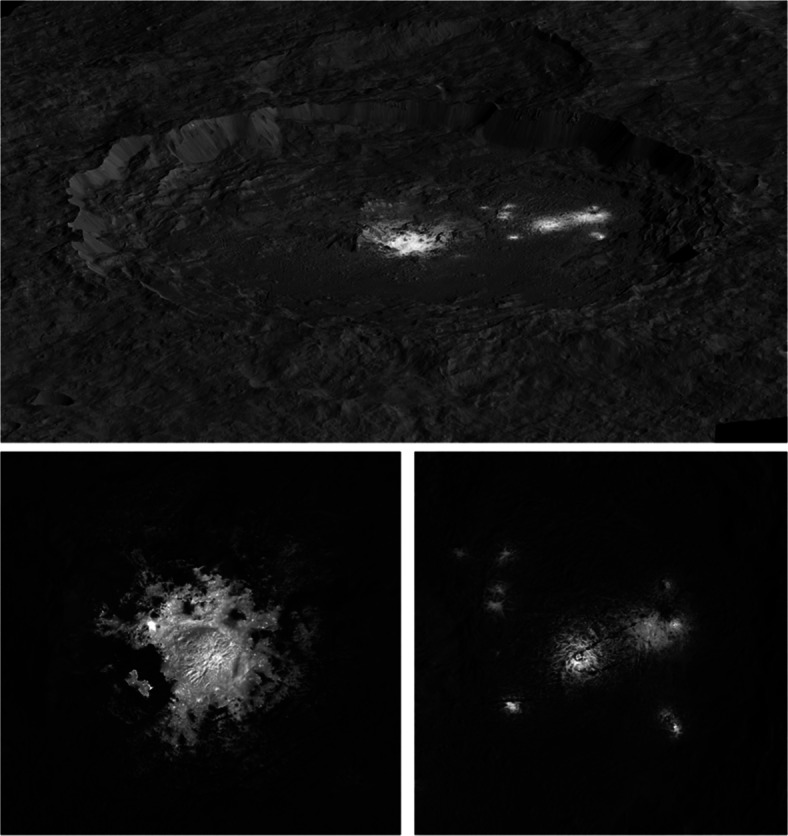
Top: Perspective view of Occator crater from the south and the bright deposits from Dawn Framing Camera data. The Cerealia Facula is saturated in this brightness stretch. Bottom left: Zoom-in view of the Cerealia Facula at the center of Occator crater shows the details of the bright deposit. Bottom right: Zoom-in view of the Vinalia Faculae (image credit: NASA/JPL/Caltech/IAPS/MPS/DLR/INAF/ASI)

The distinctive bright regions within Occator crater are one of the most remarkable features on Ceres observed by Dawn (Fig. 5). Occator crater is about 90 km in diameter, hosting the bright deposits covering the pit-dome complex named Cerealia Facula in the center, and a group of secondary bright deposits named Vinalia Faculae on the east side of the crater floor. While Ceres’ average surface contains Mg- and Ca-carbonates and Mg- and NH_4_-phyllosilicates, Occator’s faculae contain Na-carbonate, Al-phyllosilicates, and NH_4_-chloride [86]. Theoretical modelling suggested the possibility of a brine reservoir beneath Occator crater, and the gradual freezing of this reservoir was the driver of briny lavas [84]. The salts lower the eutectic point and extend the duration of volcanic activity on Ceres. Laboratory studies suggest that a slow freezing process (< 30 K/min) of the exposed ammonium-sodium-carbonate-chloride-rich brines is most compatible with the observed composition of brines observed in Occator crater [112]. A variety of morphological features are observed in the crater. Linear and concentric fractures on the crater floor are associated with cryomagmatic intrusion, and degassing or desiccation processes for the volatile rich Occator ejecta [10]. The cross-cutting relationship between stratigraphic units indicate that the Cerealia Facula was emplaced in multiple episodes [106]. Crater counting in the Occator ejecta yields an age of about 20 Myr [75], while the age of faculae is only a few Myr [73, 74].

The presence of water ice on the surface of Ceres, as well as the widespread distribution of shallow subsurface water has been associated with the active outgassing of Ceres previously observed from Earth orbit or the Earth-Sun L2 Lagrange point [1, 59]. However, the observed surface water ice exposures on Ceres do not appear to be sufficient to supply the observed water production rate [60, 61, 102]. Multiple attempts to detect water outgassing around Ceres failed, suggesting that water outgassing from Ceres is variable but also does not appear to depend on heliocentric distance [93]. Variation in the amount of water ice was detected on the wall of the Juling crater, indicating the possible existence of a seasonal water cycle [85]. Another hypothesis proposes that the solar energetic particles might be a driver of outgassing [116]. The water loss mechanisms on Ceres and the characteristics of its transient water exosphere are therefore still uncertain.

Gravitational data and geophysical modelling suggested that Ceres is partially differentiated to a ∼40 km thick and strong crust composed of rock, ice, salts, and/or clathrates with no more than 30% water ice [5, 30, 34]. Below the crust is a denser rocky mantle with a relatively weak upper layer potentially with brine-filled pore space that controls the global shape of Ceres [34]. The possibility of a dehydrated rocky core below 100 km cannot be ruled out [54].

VIR first detected an organic absorption feature at 3.4-μm on Ceres. This signature is diagnostic of organic matter and is mainly localized on a broad region of ∼1000 km^2^ close to the ∼50 km Ernutet crater [25]. The shape of the 3.4-μm band and the lack of an associated 3.25-μm feature could exclude organics with a high content of aromatic carbon such as anthraxolites as main carriers of the features on Ceres, in favour of hydrocarbons rich in aliphatic carbon [25]. Laboratory studies have found organic-rich analogues that could reproduce absorption bands in the VIR spectra [117]. However, the exact nature and concentraton history of the organic matter is still unclear, making this a compelling scientific question for any future space mission to Ceres [117]. Furthermore, organic compounds possibly also exist on top of Cerealia Facula, the brightest spot located roughly in the middle of crater Occator [26].

The volcanism and hydrothermal activity in the recent history of Ceres, the existence of brines on a global scale at the present suggest an active planetary body that could have strong astrobiological significance. The occurrence on Ceres of ammonia-bearing hydrated minerals, water ice, carbonates, salts, and organic material, revealed a complex chemical environment, potentially favourable to prebiotic chemistry in a subsurface aqueous environment [25]. The ability to detect, determine, and quantify any organics on Ceres is a clear step toward assessing habitability [16].

**Fig. 6:**
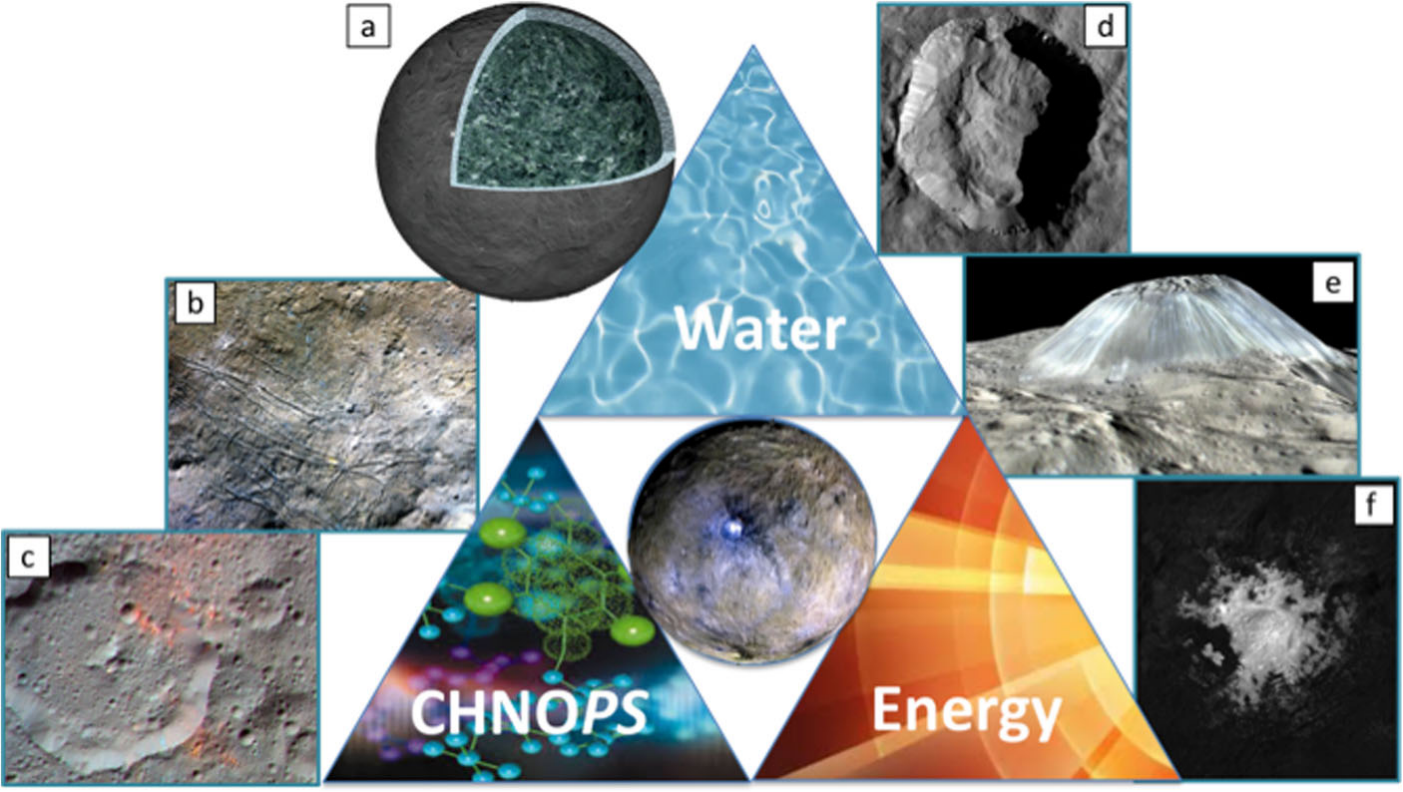
Summary of Dawn’s observations of Ceres addressed in the text. (a) Geophysical data confirmed the abundance of water ice and the need for gas and salt hydrates to explain the observed topography and crustal density. (b) Various types of carbonates and ammonium chloride have been found in many sites across Ceres’ surface (e.g., salts exposed on the floor of Dantu crater). (c) Ernuter crater (∼52 km, above) and its area present carbon species in three forms (reduced in CxHy form, oxidized in the form of carbonates and intermediate as graphitic compounds). (d) Ceres shows extensive evidence for water ice in the form of ground ice and exposure via mass wasting and impacts (Left: Juling crater, ∼20 km). (e) Recent expressions of volcanism point to the combined role of radiogenic heating and low-eutectic brines in preserving melt and driving activity (Left: Ahuna Mons, ∼4.5 km tall). (f) Impacts could create local chemical energy gradients in transient melt reservoirs throughout Ceres’ history (Left: Cerealia Facula, ∼14 km diameter). (Figure reprinted from [16]; credit for individual images: NASA/JPL/Caltech/IAPS/MPS/DLR/INAF/ASI)

Dawn findings have placed Ceres among Solar System “ocean worlds”, bodies that host current liquid ocean (not necessarily global, according to [44]). Originally classified as a “candidate” ocean world in NASA’s Roadmap to Ocean Worlds [44], Ceres is now suggested as a “real” ocean world in the light of latest results from analyses of Dawn data acquired during its extended mission ([13] and references therein), noting however that the occurrence of liquid may be limited to local or regional reservoirs. In summary, as illustrated in Fig. 6, Dawn revealed the great scientific significance of Ceres:

- Rich in water ice and other volatiles relevant to our understanding of the history of volatiles and organic matter in the inner Solar System
- Formation and evolutionary history representative of ice-rich objects in the outer Solar System
- Geologically active with minerals present only on Earth and Enceladus
- Closest and most accessible dwarf planet with volcanism and hydrothermal activity
- Ammonia-bearing hydrated minerals, water ice, carbonates, salts, and organic matter make a complex chemical environment that could favour prebiotic chemistry

### The need for Ceres samples

Vesta has now joined Mars and the Moon as the best understood extraterrestrial bodies, due at least in part to the fact that we have samples of all of them to study in the laboratory. Ceres can join that group with a future sample return.

Some exploration and scientific arguments for a Ceres sample return are:

- Although carbonaceous chondrites provide the best analog for Ceres [69], we have no meteorites from Ceres. The value of having samples for proper calibration of flight instruments and rigorous interpretation of remote sensing data is illustrated by studies of Vesta, Mars, and the Moon [51].
- Global spectral mapping of Ceres by Dawn’s VIR demonstrates that its surface is covered almost everywhere by the same assemblage (ammoniated clay, serpentine, carbonate, and a darkening agent), but in slightly differing proportions [3]. Thus, a representative regolith sample can be collected from nearly any location on the surface.
- The ice table begins below several meters depth near the equator, and approaches the surface at higher latitudes [81]. Sampling the regolith in the equatorial region would thus not require cryogenic collection and return.
- We have never before sampled an ocean world, and Ceres could be the most reachable target. There is geophysical evidence for Ceres being an ocean world [16], and our understanding of its alteration is based on the detection of a few minerals. Other phases in minor proportions in a returned sample could constrain the conditions of alteration. It is also likely that Ceres’ regolith contains some amorphous phases that could not be characterized by remote sensing.
- There is presently some controversy about the extent to which Ceres’ surface has been contaminated by exogenic chondrite impactors. These could be recognized and quantified by petrologic examination of a returned sample.
- Ceres’ chronology is based on crater size distribution analysis, which is dependent on the reference impactor flux [66], leaving the absolute ages uncertain. Radiometric dating of a sample from a mapped geologic unit [121] could help anchor Ceres’ chronology and the impactor flux in the asteroid belt.
- At several locations on Ceres, notably Ahuna Mons and Occator Crater, brines have recently erupted and deposited salts (sodium-carbonate, ammonium chloride, plus more phases that have not been identified [12, 24, 113, 114, 123]. Small quantities of these phases, which provide important constraints on the nature of subsurface fluids, may be present in regolith samples close to such outcrops.
- Organic matter discovered at one location on Ceres [25] suggests that it should be widely distributed in lesser amounts. The presence of carbon all across Ceres’ surface with abundances greater than in CI chondrites suggests a widespread water alteration process associated with organic chemistry [67, 82]. Understanding the organic component on Ceres has important implications for prebiotic chemistry and astrobiology.
- We do not know whether Ceres formed near its present location in the asteroid belt or in the giant planet region and was later scattered into the main belt by giant planet migration and resonances. Measurement of isotopes of H, C, and N will place constraints on the origin of water and organics. Isotopes of oxygen, chromium, titanium, etc. [104] can place Ceres into its proper formation setting, as it has for other bodies for which we have samples.
- Comets from the outer Solar System have long been suspected as the source of Earth’s water [78]. However, while a recent deuterium-to-hydrogen (D/H) ratio measurement for a comet is compatible with terrestrial ocean water [42], most comet D/H measurements are not [2]. Meanwhile, dynamical studies [76] indicate that large quantities of Earth’s water could have been supplied by objects from the region of the Solar System coinciding with the present-day outer asteroid belt. If Ceres can be determined to have formed in situ, a D/H ratio (in addition to other isotopic ratios) measured for Ceres would help to assess the plausibility of the main asteroid belt as a source of the terrestrial water that is so critical to the rise of life on Earth.

## Mission scenarios

### Overview

If we follow the long-term trend in the development of scientific missions to the Moon and Mars, respectively, and that of asteroidal exploration, we would probably reach the conclusion that they might likely converge on a large-scale international space program of Ceres. NASA’s Dawn mission has yielded many exciting results, but the scientific observations were limited to just three remote-sensing instruments, namely the framing camera, the visible and near-infrared mapping spectrometer, the gamma-ray spectrometer/neutron detector, as well as gravity science. A more comprehensive payload on future orbiter(s) is needed for a full characterization and understanding of the surface and atmospheric/exospheric environment of Ceres. In addition, just like in the case of lunar or Mars exploration, different types of platforms such as lander and rovers might be required to address astrobiology objectives. A sample return mission might be regarded as the final step. Furthermore, the water- and organic-rich environment of Ceres makes it an attractive candidate location for future research stations in support of deep space exploration.

**Fig. 7:**
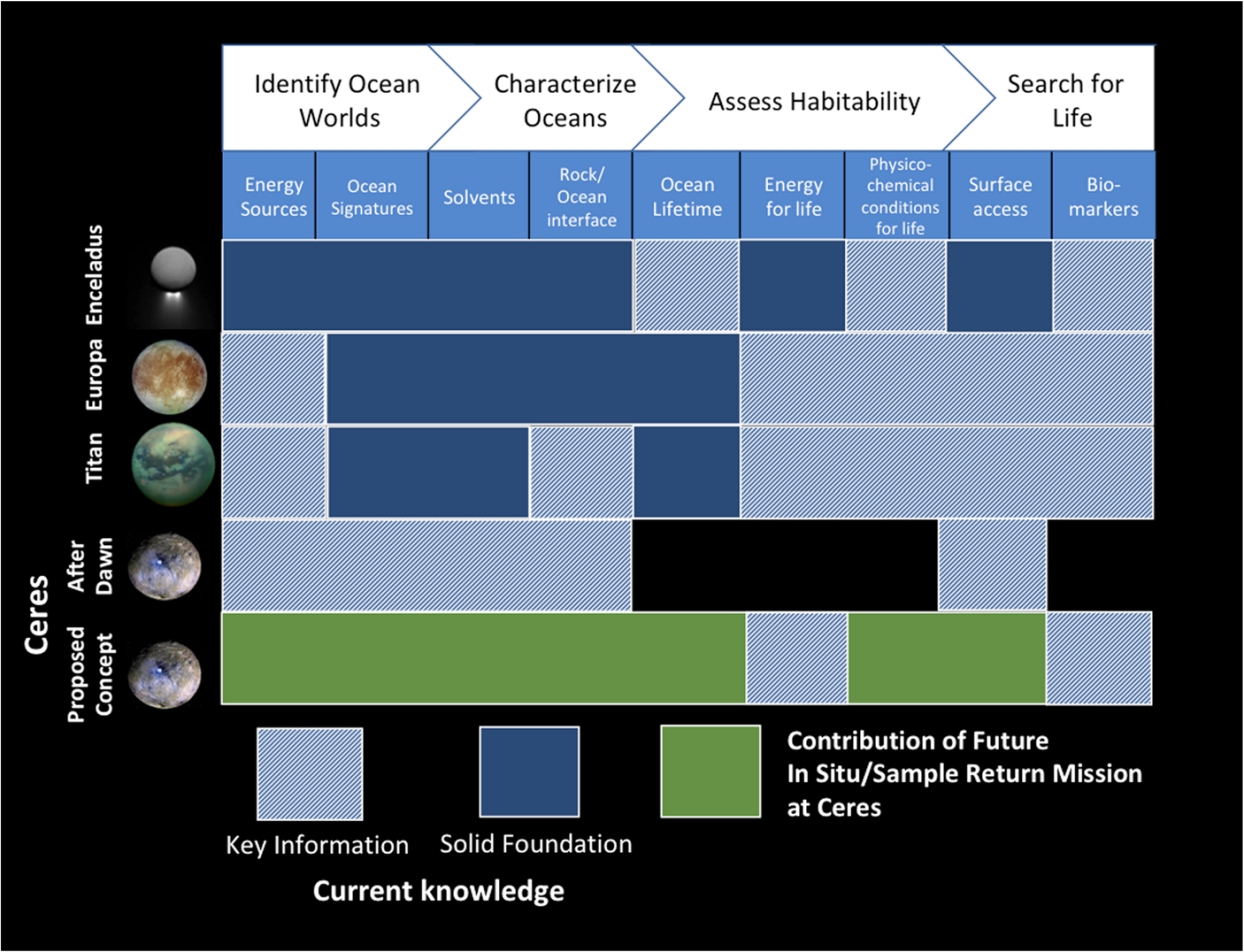
The contribution of future Ceres in-situ and sample return mission to the exploration of the Solar System ocean worlds and their habitability (Based after [44])

As implied by “Dawn”, the name of the first mission to Ceres, and the name of the present mission proposal “GAUSS”, the in-depth exploration of Ceres should be viewed in terms of detailed investigations of the genesis and evolution of the asteroid belt and the Solar System. The contribution of future in-situ and sample return exploration of Ceres in the context of studying the Solar System ocean worlds and their habitability is illustrated in Fig. 7. It can therefore be envisaged that besides orbiter observations, a set of lander(s) and rover(s) would be needed. It is also possible that this program can be composed of a series of missions over several decades to address different specific scientific questions.

Even though our main focus is to initiate the planning of an ESA-L-class-level sample return mission, it could be streamlined to a Rosetta/Philae lander style mission for cost reasons and expediency. The cost envelope might hence be fitted within the budget of an M-class project depending on the mission component to be chosen. Similar consideration would probably be pursued by CNSA also if it agrees to support a joint assessment study. It is important to emphasize that a sample return mission might still be possible with the participation of additional national agencies, besides CNSA and ESA as postulated here. Possible mission scenarios are summarized in Table 1.

**Table 1:** Summary of mission scenarios

Mission type	Mission component	Mission class
Sample return	Orbiter+lander+return capsule	L-class, or M-class with significant contribution from CNSA
Landing/Roving	Orbiter+lander / hopper / rover	M-class with possible lander/rover contribution by CNSA

In the scenario of a cryogenic sample return mission, GAUSS will first perform a high-resolution global remote sensing investigation, characterizing the geophysical and geochemical properties of Ceres. Candidate sampling sites will then be identified, and observation campaigns will be run for an in-depth assessment of the candidate sites. Once the sampling site is selected, a lander will be deployed on the surface to collect samples and return them to Earth in cryogenic conditions that preserve the volatile and organic composition as well as the original physical state enabled by state of the art technologies.

The scientific goals and related measurement objectives of Project GAUSS are summarized in the traceability matrix (Table 2). The four scientific goals focus on understanding: the origin of Ceres, its evolution and current state, its habitability, and its connection to the carbonaceous meteorite collections. The results from these investigations will have direct implications on our understanding of the evolution of the Solar System and in particular of the icy satellites.

**Table 2:** Traceability matrix for Project GAUSS

Scientific goal	Measurement objective	Instrument
The origin and transportation of water and other volatiles in the inner Solar System: Where does Ceres come from?		
Ceres chronology	Crater counting, radiometric dating of samples	Sample analysis, camera, topographic camera
Connection between Ceres composition and pre-solar materials	Stable isotopes of oxygen, chromium, titanium	Sampling mechanism, microscopic camera
Volatile inventory on Ceres	Surface and subsurface water, other volatile species including Na, K, S, Cl, etc.	Sampling mechanism, Near-Infrared spectrometer (NIR), Thermal Infrared spectrometer (TIR), Active Particle-induced X-ray Spectrometer (APXS), Gamma Ray Spectrometer (GRS)
Contamination from exogenous materials on Ceres and their roles in the evolution of Ceres	Surface mineralogy, petrological units in samples	Sampling mechanism, camera, NIR, TIR, microscopic camera
Physical properties and internal structure of Ceres: What do ice dwarf planets look like?		
Structure of the near-subsurface and deep interior, and the implications to the differentiation and aqueous alteration processes of Ceres	Surface mineralogy, elemental abundance, gravity	Imaging, NIR, TIR, seismology, radar, subsurface science package
Geological processes, in particular current and past cryovolcanism	Topography and morphology, gravity	Imaging, lidar, radar, radio science, seismometer
The existence and characteristics of Ceres’ exosphere	Gas species around Ceres	Gas chromatography-mass spectrometer (GC-MS), Ion and mass spectrometer, UV spectrometer, dust detector
The astrobiological implications of Ceres: Was it habitable in the past and is it still today?		
Existence of liquid water inside Ceres, its extent, distribution, and depth	Topography, morphology, gravity	Camera, radar, lidar, topographic camera, radio science, seismometer, subsurface science package
Redox condition on Ceres, the existence and forms of oxidants	Elemental abundance, forms, and isotopic ratios of O, S, Cl, N	Sampling mechanism, NIR, TIR, APXS, GRS
Abundance, sources and sinks, and chemical forms of life-forming elements; source of terrestrial water	Elemental abundance, forms, and isotopic ratios of C, H, N, O, S	Sampling mechanism, NIR, TIR, APXS, GRS
Inventory and composition of organic compounds, their origins and evolutions	Existence, abundance, and types of organic materials	Sample mechanism, NIR, TIR
Mineralogical connection between Ceres and the collections of primitive meteorites: Where are Ceres meteorites?		
Thermal metamorphism and aqueous alteration history of Ceres	Mineralogy, petrological characterization, isotope ratios	Sampling mechanism, NIR, TIR
Fractionation of elements and the geochemical processes	Mineralogy, elemental abundance, isotope ratios	Sampling mechanism, NIR, TIR

### Candidate sites for in-situ investigation and sample return

**Fig. 8:**
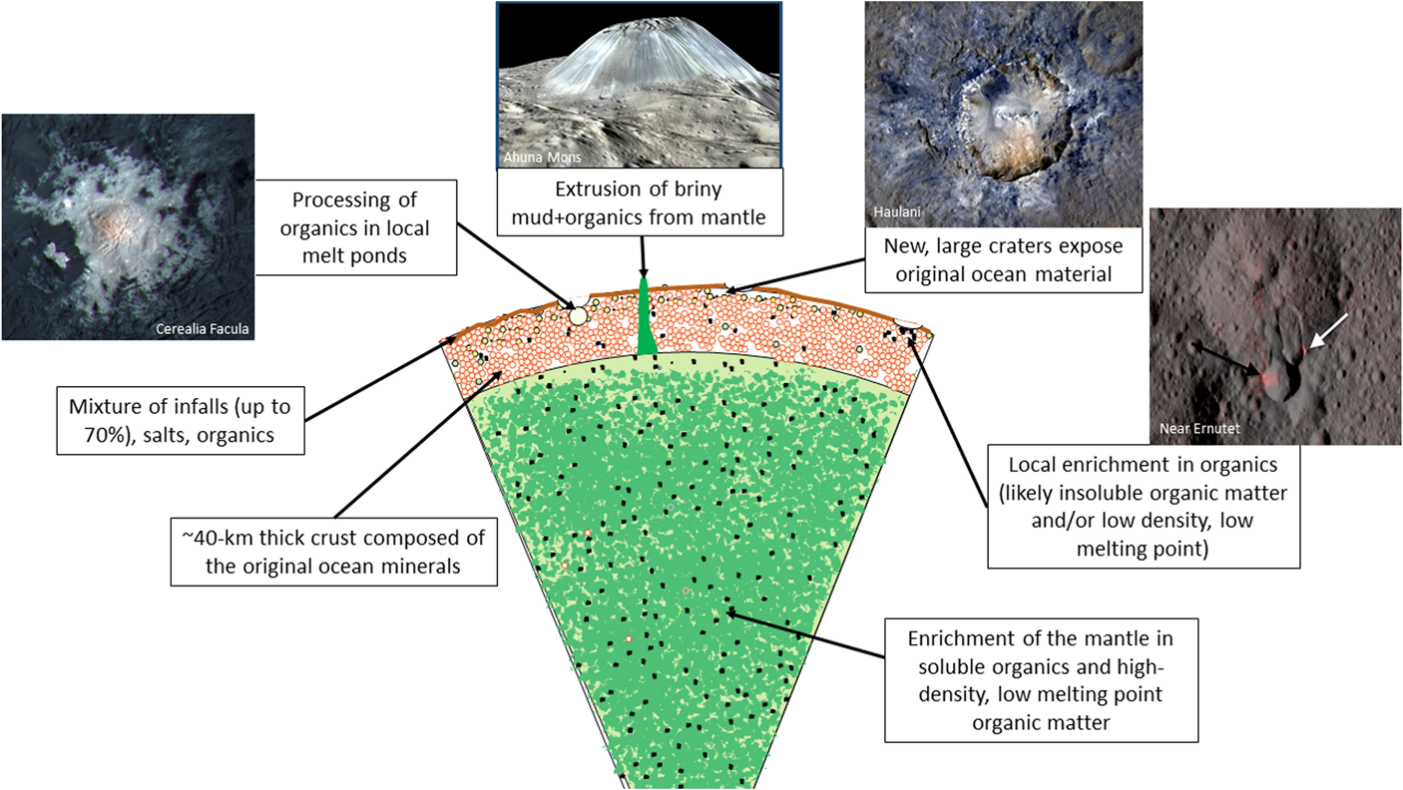
Surface geomorphological features on Ceres and their connections to the interior structure (image credit: NASA/JPL/Caltech/IAPS/MPS/DLR/INAF/ASI)

**Fig. 9:**
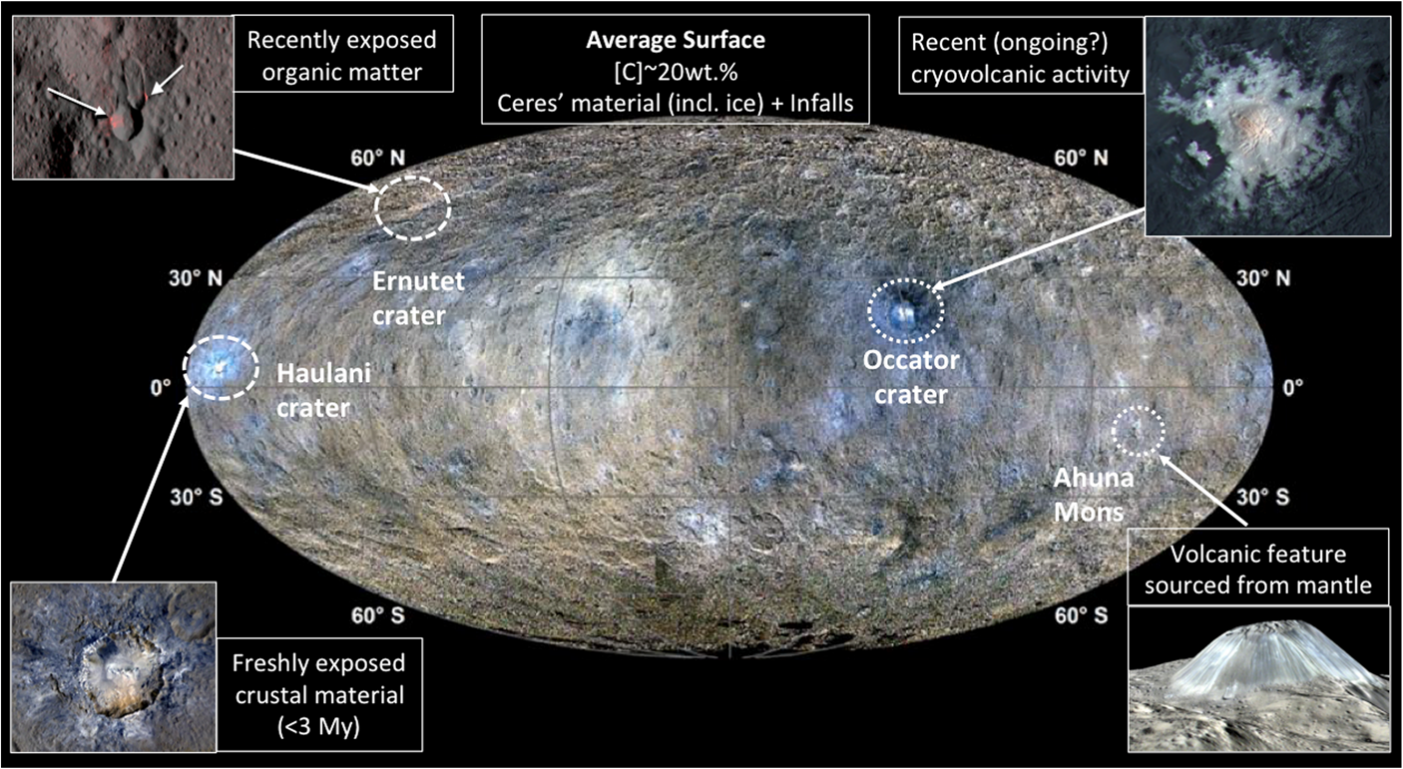
Candidate sites on Ceres for in-situ and/or sample return. The Ceres basemap is a composite color map of Ceres generated by Dawn Framing Camera images with R, G, B = 960, 550, 440 nm, respectively (from Castillo-Rogez et al, Pre-decadal survey, image credit: NASA/JPL/Caltech/IAPS/MPS/DLR/INAF/ASI)

The geomorphological features on the surface of Ceres are connected to its interior and formed through recent geological processes (Fig. 8). The mantle of Ceres is composed of hydrated minerals, and may be enriched in organics and high-density, low melting point organic matter in localized areas. The ∼40 km thick crust above the mantle is composed of the original ocean materials, salts, carbonates, and brine. Brine reservoirs existed in the recent past and drove volcanic activity. Ahuna Mons might be an extrusion feature of briny mud and organics from the upper mantle [95]. The surface layer of crust is covered by a mixture of infalls, salts, and organics. Cerealia Facula is covered by salts that are left behind after subsurface brine was accessed by impact-produced fractures and reached the surface through the cracks and eventually evaporated [87]. The Haulani crater is one of the youngest impact craters, associated with bright blue rays of ejecta that are freshly exposed crustal materials and could represent the original ocean materials [113].Therefore, the Dawn observations of Ceres indicate these sites carry profound scientific implications serving the scientific goals identified for Project GAUSS for in-situ investigations and sample returns (Fig. 9).

### Proposed payload

Only three instruments and gravity science were carried by Dawn. A more comprehensive payload would be needed to characterize Ceres itself and its atmospheric and space environment. This is especially true with the detection of water plume activity by Herschel [59]. Similar to the Cassini measurements at Enceladus, repeated fly-through of the gas plume would allow the identification of the chemical composition and isotopic ratios of the gas molecules, thus providing a probe to the nature of the subsurface lake/ocean. The proposed payload and their heritage are summarized in Table 3. It is assumed that some of the scientific instruments will have been successfully developed for CNSA’s Tianwen-1 mission to Mars and the China Asteroid Explorer (CAEX) to small bodies. By the same token, the sampling and reentry technology are also assumed to be derived from China’s sampling missions to the Moon, Mars, and asteroids. The instrumentation on the Philae lander on ESA’s Rosetta, the Rosalind Franklin Rover (RFR) of ExoMars, the Yutu rovers of Chang’e missions, and the Zhurong rover of the Tianwen-1 mission, will provide strong preparation and heritage for the Ceres rover.

**Table 3:** Strawman payload on the Orbiter and the Lander

	Payload name	Heritage
Orbiter	Wide-angle and narrow-angle camera	Chang’e 1-3, Dawn, Rosetta, BepiColombo
	Infrared imaging spectrometer	Dawn, Rosetta, Chang’e-4, CAEX
	Fourier Transform Spectrometer	Mars Express, ExoMars
	Thermal mapper	CAEX
	Ultraviolet imaging spectrometer	Chang’e-3, MMX
	GRS	Change’e 1-2
	Long-wavelength radar	CAEX
	Lidar	BepiColombo
	Dust detector	Rosetta, CAEX
	Ion and mass spectrometer	Rosetta, CAEX
	Particles and Fields package	Rosetta, CAEX
	Radio Science	Rosetta
Lander	Topographic camera system	Chang’e 3-4; Mars rovers; Philae
	Microscopic camera	Philae, CAEX, CLUPI (ExoMars)
	APXS	Chang’e 1-3
	GRS	Chang’e 1-2
	Laser-induced breakdown spectroscopy	Tianwen-1, Curiosity
	GC-MS	Philae, CAEX
	Subsurface science package	Philae, CAEX
	Active seismometer	Insight, Chang’e 7

It is important to emphasize the astrobiological significance of the in-situ exploration of geological landmarks of special interest. These include the Ernutet crater and the Occator crater. To achieve high confidence in detecting organic materials on the surface of Ceres and assess their nature, the mid-IR range 2-12μm turns out to be crucial at the spatial resolution that could be achieved by a spacecraft in orbit around Ceres, not necessarily with imaging capabilities. Covering this sensitivity range would allow:

- Complementing surface mineralogy as derived by VIR in the overall 0.4-5.1 μm spectral range by using thermal emission spectroscopy from 2-12 μm, determining the specific class of compounds responsible for the organics-rich area observed close to crater Ernutet, and shedding light on its origin (endogenous vs. exogenic). This spectral range would ultimately provide the capability to greatly expand our inventory of astrobiologically important compounds, and to remotely sense complex organics.
- Assess any potential ongoing activity and occurrence of organics, particularly on top of Cerealia Facula in crater Occator.
- Perform an in-depth characterization of the 34-km crater Haulani, one of the youngest geologic features on Ceres and home to the most prominent thermal signature observed on the entire surface of the dwarf planet [113, 114].
- Retrieve surface temperatures, grain size, porosity and surface roughness using thermal emission from 2-12 μm, thus accessing temperature values below 180 K with high accuracy, which were precluded to Dawn/VIR.
- Ultimately characterize the best landing site for a surface element in terms of composition and roughness (regolith depth).

The Fourier Transform Spectrometer (FTS) working principle is based on the Michelson interferometer, which is an infrared spectrometer suitable for covering a broad spectral range from the near infrared to the mid infrared with constant, high spectral resolution (typically up to 1-2 cm^-1^). FTSs simultaneously detect light varying the optical path difference (OPD) and encoding the signal at each wavelength with a cosine modulation at a frequency proportional to the wavenumber *ν*= 1/*λ*. This interferogram is then transformed into a spectrum, i.e., from the optical path domain to the optical frequency domain. The OPD could be produced by rotating a double pendulum system around its axis, rather than translating the moving mirror along a linear direction like in the classical Michelson interferometer. Compared to the first generation of FTS (e.g. PFS on board the ESA Mars Express spacecraft), in recently upgraded versions have been proposed that offer a significant reduction in mass and size, adopting innovative technical solutions.

### Trajectory design

**Fig. 10:**
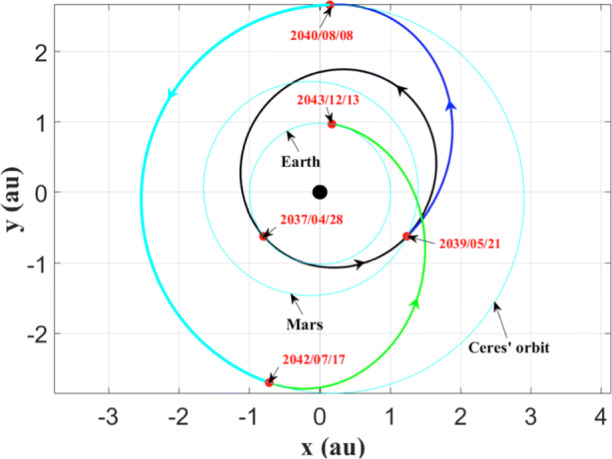
The transfer trajectory for Ceres’ sample return mission. In the transfer scenario, a Mars’ gravity assist is taken into consideration. The probe launches from the Earth on April 28th, 2037. The arc from the Earth to Mars is shown in black line, the arc from Mars to the target asteroid is given in blue line, the rendezvous segment is presented in cyan line, and the return trajectory from the asteroid to the Earth is marked in green line. The critical points of time are represented by red dots

Considering the fact that the target asteroid is located between the orbits of Mars and Jupiter (the semi-major axis of Ceres is about 2.768 au), in the transfer design we take advantage of a gravity assist of Mars in order to reduce the required fuel consumption. In the preliminary transfer scenario, the sequence is given as follows: (a) launching from the Earth; (b) Mars’ gravity assist; (c) rendezvous with Ceres; (d) returning to the Earth. The launch window is assumed to be after January 1st, 2035. The planar transfer trajectory is reported in Fig. 10 and the associated parameters are provided in Table 4.

**Table 4:** Parameters of the interplanetary trajectory for the Ceres sample return mission

Time points		Velocity impulse	
Launch	April 28, 2037	Hyperbolic excess velocity ($V_{\infty }$)	4.961 km/s
Mars’ gravity assist	May 21, 2039	Deep space maneuver at perimartian (Δ*V*_*M*_)	1.981 km/s
Rendezvous with Ceres	August 8, 2040	Braking velocity for rendezvous with Ceres (Δ*V*_*f*_)	5.400 km/s
Departure from Ceres	July 17, 2042	Accelerating velocity for departing from Ceres	4.813 km/s
Earth re-entry	December 13, 2043		

### Technological developments required

Here we discuss the key technological requirements and main challenges for a Ceres sample return mission.

#### Flight dynamics

It is assumed, that by the time of the Voyage 2050 programme, the capacity of launch vehicles poses no challenge for sending a spacecraft with a lander and re-launch system to Ceres. Ceres does not have an atmosphere that exhibited any detectable dynamical effects on the Dawn spacecraft at altitudes down to 35 km. Therefore, an atmospheric entry system is not needed to land on Ceres. The descent and landing system for Ceres can be derived from the already mature Chinese Chang’e 3 and 4 descent and landing system. The technology for a re-launch system already exists since the Apollo era, and a similar system is already in development for the various Mars sample return missions under study (see Visions and Voyages, the Planetary Science Decadal Survey 2013-2022 report, National Research Council of the National Academies). The escape velocity from Ceres’ surface of about 0.5 km/s is much lower than that of the Moon (2.4 km/s) and Mars (5 km/s). Therefore, the re-launch system from Ceres’ surface for sample return can be much smaller and light-weight than those for the Apollo system (which also had a complicated life-support system) or any Mars sample return system in development. It can be expected that the needed technology will have been developed and matured by the 2030-2040 timeframe, and should be much more efficient (small and light-weight) than the currently available system and hence will require a less powerful launch vehicle than required by present technology. Earth re-entry is already mature and is not expected to pose any challenge. In addition, an ion propulsion system, like the one used by the Dawn spacecraft should be much more efficient.

#### Sampling on Ceres

One or more approaches need to be developed for sampling the surface layer of Ceres. Sampling on Ceres is different from that on the Moon or Mars, which have relatively strong surface gravity and don’t require an anchoring mechanism. Ceres sampling would also be different from that on small asteroids such as the targets of JAXA’s Hayabusa and Hayabusa 2, and NASA’s OSIRIS-REx, which have loose regolith and micro-gravity allowing for a touch-and-go approach. With its surface gravitational acceleration of ∼0.27 m/s^2^, a robust anchoring mechanism may be required for sampling on Ceres, depending on the sampling system. A drilling system may also be needed to drill into the relatively strong rock-ice-salt mixture with a density of ∼1.3 g/cm^3^ [29]. A drilling depth of decimetres, but less than one metre is likely needed in order to reach the water-ice rich layers or subsurface ice table at high latitudes [81]. Technologies currently being developed for landing on and sampling asteroids might provide the necessary heritage for project GAUSS, e.g., technologies being developed for the Chinese small body mission [125]. The various sampling mechanisms and collection system for Mars sample return currently in use [65, 122] are good references for the future development of a Ceres sample return system. Note that ESA and NASA signed a statement of intent on Mars sample return.

#### Cryogenic sample collection, containment, and curation

The idea of cryogenic sample return from icy Solar System bodies has been around for a long time. The Rosetta mission was originally designed to return samples from a comet at cryogenic temperature [49]. This is probably the most challenging part of the entire project. To preserve the volatile and organic compounds in their original status, samples should not be thermally or aqueously altered during the collection, storage and transport, and they should be shielded from contamination by terrestrial volatiles upon return. Therefore, the samples need to be collected and sealed in the containers under cryogenic temperature and overpressure. A temperature of <∼170 K is required, and the original temperature of subsurface samples (probably ∼140 K; [43, 99]) is desired, throughout the entire collection, return, and curation process for preserving water ice. The sample return capsule should have a number of separated and individually sealed containers that can be overpressured to prevent the loss of volatiles such as H_2_O, HCN, S-bearing species, and cyanides [17, 18].

### Planetary protection

As revealed by the Dawn observations, the surface of Ceres is organic rich, with certain areas exhibiting higher concentration (e.g. the Ernutet crater; [25]). Furthermore, multiple regions on Ceres show signs of recent or even ongoing brine-driven activity that could expose fresh materials from Ceres’ interior. Although it is unlikely that the organic matter on Ceres is of biological origin, it is required to assess planetary protection strategies for any future lander or sample return mission to Ceres. Based on the current guidelines for planetary protection [39] and synthesized analyses of the Dawn results, we summarize in Table 5 preliminary considerations on planetary protection for different types of mission to Ceres.

**Table 5:** Planetary protection categories for different types of missions to Ceres

Mission type		Category	Note
Lander/Rover	Most part of the surface	II*	Like Dawn, the mission is at least Category III if it includes a Mars gravity assist.
	Occator faculae	IV	
Sample return	Different possible sites, including organic-rich areas	V	Determination on the sub-category of being “restricted” or “unrestricted” depends on location.

Further information to be found in Castillo-Rogez, J. C. et al. “Planetary Protection Requirements for Future Exploration of Ceres–State of Understanding after the Dawn Mission” (in revisions)

## International context

We note that the ESA roadmap to Ceres needs to be constructed within an international context. NASA has recently selected in situ exploration and sample return concepts at Ceres to be studied in preparation for the 2023-2032 planetary science decadal survey [14]. That study will cover a range of architectures with a focus on long-range mobility, sample acquisition, and return to Earth. CNSA has also expressed interest in a mission to Ceres beyond its exploration of the Moon, Mars and small bodies [127]. It is, therefore, essential for ESA to explore cooperative opportunities through joint assessment studies with international agencies.

This mission concept came about during a round-table discussion at the 4th Lunar and Deep-Space Exploration International Conference between July 22 and 24, 2019, in Zhuhai, China. One important objective of this proposal is therefore to promote scientific cooperation of the Chinese planetary science community with its European counterpart, taking advantage of the momentum of CNSA and the spirit of ESA’s “Voyage 2050 Initiative”. It might begin with a joint assessment study co-sponsored by both agencies or their representatives. The assessment should identify the scientific objectives, technical requirements, mission architecture, and possible division of responsibilities and authorities.

The extensive orbital measurements of the Dawn mission can be used as a robust basis for planning this large scale mission. Because of the efforts of several agencies including JAXA, NASA, and ESA over the last two decades, the exploration of asteroids has become a major component of deep space missions. On the one hand, sample return missions and the related technologies have been championed by JAXA that have successfully executed the Hayabusa mission to the S-type asteroid Itokawa and the Hayabusa2 mission to the C-type asteroid Ryugu. This line of approach will be extended to a sample return mission (Mars Moon Explorer or MMX) to the Martian moon Phobos and likely a sample return mission to a Jovian Trojan asteroid [77]. Following the Dawn mission, NASA has demonstrated its strong interest in asteroid in-situ exploration by performing the OSIRIS-REx sample-collection project to the B-type asteroid Bennu, and developing two more asteroid rendezvous missions, one to the large M-type asteroid 16 Psyche, and the other one (Lucy) to a number of Jovian Trojans (flybys). The Chinese space agency, CNSA, has recently issued an AO calling for instrument proposals and international cooperation for its first asteroid sample return mission to a near-Earth asteroid, followed by a rendezvous mission with a main-belt comet [126]. There is also an ongoing assessment study for a sample return mission to an inner-belt E-type asteroid. In parallel to this heightened level of activities by JAXA, NASA and CNSA, ESA has approved the daring “Comet Interceptor” project on the heel of the Rosetta/Philae mission.

If the above set of planned (or proposed) space projects can be successfully carried out, then the first phase of reconnaissance/sample-return missions to major phenotypes of small asteroids and short-period comets–as a global enterprise–would be completed by 2030-2035. It is also expected that the basic knowledge gained and technologies introduced would be applied to the next phase of asteroidal exploration–beginning around 2030, if not earlier, with the goal to address the need of planetary defense against asteroid impact hazard, and asteroidal mining for commercial reasons.

The lunar samples returned by the Apollo missions half a century ago continue to spur cutting-edge science. We believe that Ceres should be viewed as the next frontier space laboratory to extend our knowledge of the Solar System much further beyond.

### The GAUSS Team (continued)

John Carter (Institut d’Astrophysique Spatiale), Grégoire Danger (Aix Marseille Univ, CNRS), Julia de Leon (Instituto de Astrofísica de Canarias), Jörn Helbert (DLR Institute of Planetary Research), Xiyun Hou (Nanjing University), Hauke Hussmann (DLR Institute of Planetary Research), Katherine Joy (University of Manchester), Tomas Kohout (University of Helsinki), Alice Lucchetti (INAF-Astronomical Observatory of Padova), David Mimoun (Université de Toulouse), Olga Muñoz (Instituto de Astrofisica de Andalucia), Jose Luis Ortiz (Instituto de Astrofisica de Andalucia), Maurizio Pajola (INAF-Astronomical Observatory of Padova), Antti Penttilä (University of Helsinki), Frank Preusker (DLR Institute of Planetary Research), Ottaviano Ruesch (University of Münster), Pablo Santos-Sanz (Instituto de Astrofisica de Andalucia), Nico Schmedmann (University of Münster), Nicole Schmitz (DLR Institute of Planetary Research), Katrin Stephan (DLR Institute of Planetary Research), Guneshwar Thangjam (NISER), Josep M. Trigo-Rodríguez (Institute of Space Sciences, CSIC-IEEC), Cecilia Tubiana (INAF-IAPS), Vassilissa Vinogradoff (Aix Marseille University, CNRS), Liangliang Yu (Macau University of Science and Technology), Francesca Zambon (INAF-IAPS), Yuhui Zhao (Purple Mountain Observatory, CAS)
